# Predictive Intelligent Transportation: Alleviating Traffic Congestion in the Internet of Vehicles

**DOI:** 10.3390/s21217330

**Published:** 2021-11-04

**Authors:** Le Zhang, Mohamed Khalgui, Zhiwu Li

**Affiliations:** 1 School of Intelligent Systems Science and Engineering, Jinan University, Zhuhai 519070, China; le.zhang.chinese@gmail.com; 2 National Institute of Applied Sciences and Technology, University of Carthage, Tunis 1080, Tunisia; 3 Institute of Systems Engineering, Macau University of Science and Technology, Taipa 999078, Macau; zwli@must.edu.mo

**Keywords:** traffic congestion, traffic signal control, vehicle route guidance, Internet of Vehicles

## Abstract

Due to the limitations of data transfer technologies, existing studies on urban traffic control mainly focused on isolated dimension control such as traffic signal control or vehicle route guidance to alleviate traffic congestion. However, in real traffic, the distribution of traffic flow is the result of multiple dimensions whose future state is influenced by each dimension’s decisions. Presently, the development of the Internet of Vehicles enables an integrated intelligent transportation system. This paper proposes an integrated intelligent transportation model that can optimize predictive traffic signal control and predictive vehicle route guidance simultaneously to alleviate traffic congestion based on their feedback regulation relationship. The challenges of this model lie in that the formulation of the nonlinear feedback relationship between various dimensions is hard to describe and the design of a corresponding solving algorithm that can obtain Pareto optimality for multi-dimension control is complex. In the integrated model, we introduce two medium variables—predictive traffic flow and the predictive waiting time—to two-way link the traffic signal control and vehicle route guidance. Inspired by game theory, an asymmetric information exchange framework-based updating distributed algorithm is designed to solve the integrated model. Finally, an experimental study in two typical traffic scenarios shows that more than 73.33% of the considered cases adopting the integrated model achieve Pareto optimality.

## 1. Introduction

Traffic congestion is a phenomenon in which vehicles move slowly due to traffic surges [1,2]. It costs serious direct and indirect economic losses in almost all modern cities [3]. Traffic congestion has become a major issue for city managers. Various traffic management systems and methods are proposed to alleviate traffic congestion [4,5,6,7].

Engineers usually balance the distribution of traffic flow to mitigate traffic congestion [8]. They adjust the timing or direction of traffic flow elements (such as cars, pedestrians, and bicycles) to avoid vehicular excessive concentration. The existing studies in this field can be divided into two categories: traffic signal control (TSC) and vehicle route guidance (VRG) [9,10,11,12].

TSC releases or blocks traffic flow at intersections in the time dimension by switching traffic signal between “green” and “red” [13]. The process of signal switching can be described as a nonlinear programming model [14]. For example, the work in [15] takes the minimum delay time of vehicles as an objective function and obtains the well-known *Webster Equation* for traffic signal timing. The authors in [16] take the expected travel time and standard deviation of travel times as a multi-objective function. Then a genetic algorithm is adopted to solve their TSC model. In [17], Chiou proposes a bi-level TSC model to minimize total travel time and risk exposure for transport of hazardous material at intersections.

VRG adjusts the trajectories of traffic flow in space dimension for alleviating congestion [18]. A route guidance system uses advanced positioning and computation technologies to provide drivers optimal paths based on travel requirements [19]. Selecting vehicular optimal paths can be described as an integer programming problem [11]. For example, the work in [20] takes the shortest distance as an objective function using the well-known *Dijkstra Algorithm* for path optimization. In [21], the authors add a safety index into the traditional shortest distance to propose a multi-objective VRG model, and adopt a reinforcement learning algorithm to compute the improved routes. The study in [22] is based on an energy consumption prediction model to integrate an energy index into VRG, where an improved *Dijkstra Algorithm* is used to calculate routes in real-world experiments.

The above precise calculation models of TSC and VRG are the main research topics for urban traffic engineers [8]. They use the advanced intelligent algorithms (such as a heuristics algorithm, machine learning, etc.) to solve various complex nonlinear program models based on historical traffic data. Since real-time traffic information collection is both costly and insufficient due to limited detection technologies [19], these methods can only generate fixed or segmented dynamic schemes for alleviating traffic congestion but are infeasible for the varying traffic conditions.

In recent years, the emergence of the Internet of Vehicles (IoVs) enables huge potential in the area of intelligent transportation [23,24,25,26,27,28,29,30,31]. It mainly includes vehicle-to-vehicle (V2V), vehicle-to-road (V2R), vehicle-to-infrastructure (V2I) and so on. This technique can make effective use of the real-time traffic network information through wireless sensor networks, and provides various functional services for the vehicle operation [25]. IoVs has become an intensively studied area with a multitude of articles.

For example, the work in [23] builds a smart traffic management platform to collect available real-time traffic data based on IoVs, and successfully demonstrates this system on real roads. The research in [27] builds a car-following model to propose a car-following model using vehicles to everything (V2X) technique. In [28], a way of transmitting warning messages by V2V and V2I are designed to avoid road accidents. The study in [30] designs a context-aware antenna selection model to optimize the locations of 5G antennas. The authors in [31] propose the concept of Mobile-Generated Content to enhance the production efficiency of intelligent connected vehicles in sustainable cities. With the increasingly mature of IoVs, a large amount of literature adopts this technique to address the dynamic reconfiguration of TSC and VRG [32,33,34,35,36,37].

For the IoVs-based dynamic TSC, the study in [32] combines the real-time position data from connected vehicles with intersection information from inductive loops to produce dynamic signal timing at isolated intersections. The work in [33] introduces the joint passing rates between adjacent intersections to present an adaptive multiple intersections TSC model based on the IoVs technique that enables vehicles to communicate with each other. The authors in [34] use Vehicular Ad hoc Networks to optimize TSC schemes and build a vehicular cloud computing platform.

For the IoVs-based dynamic VRG, the work in [35] uses infrastructure agents to receive vehicle route plans and recalculates vehicle paths based on vehicle-to-infrastructure communication. The authors in [36] employ Road Side Units to detect current road congestion and use Fog-Cloud computing to update vehicular route plans constantly. With the help of vehicle-to-infrastructure technique, a deep Q-network algorithm is proposed in [37] to determine autonomous vehicle routes based on fuel economy and driving safety.

The above IoVs-based methods for alleviating traffic congestion only use real-time traffic data to produce decisions in isolated dimension control of TSC or VRG. In real traffic, however, the distribution of traffic flow is the result of multi-dimension working together [38]. The decisions of each dimension can influence other dimensions’ results by feedback regulation in the whole traffic system. For example, vehicular path reconfigurations could change future distribution of traffic flow in TSC. Reversely, different signal control schemes determine the time taken for vehicles passing intersections in VRG.

No matter how efficiently the isolated dimension control is improved, it can only produce decisions without considering different dimensions’ interplay which results in the waste of city since of the non-coordinated schemes. Although the works in [38,39] attempt to combine the two dimensions using IoVs technique to alleviate traffic congestion together, their coordinated mechanism ignores the two-way feedback regulation between TSC and VRG. Specifically, they suppose that each vehicle deposits intention pheromone (route schemes) along its route, and use these data to predict future traffic densities to help signal controllers reconfiguring. Their coordinated mechanisms are designed based only on one-way regulation from vehicle routes to signal schemes.

Based on the feedback regulation between TSC and VRG and their basic models—*Webster Equation* and *Dijkstra Algorithm*, this paper proposes an integrated predictive intelligence transportation (PIT) model to optimize signal schemes and vehicle routes simultaneously by considering the future traffic coming from other dimensions’ decisions (two-way feedback regulation). Compared with the isolated-dimensional TSC and VRG control strategies, the challenges of this model lie in that the formulation of the complex nonlinear relationship between TSC and VRG is hard and the design of a corresponding solving algorithm that can obtain Pareto optimality for multi-dimension control is complex.

For the nonlinear relationship of TSC and VRG, taking the *Webster Equation* as a benchmark model, we first introduce the predictive traffic flow as a medium variable to formulate the link from the varying route plans to TSC. A predictive traffic signal control (PTSC) model considering the future traffic flow influence from VRG is built. Second, taking the *Dijkstra Algorithm* as a benchmark model, the predictive vehicular waiting time is introduced to formulate the link from different signal schemes to VRG. A predictive vehicle route guidance (PVRG) model is formulated considering the future waiting time at intersections determined by TSC. Third, the proposed PTSC and PVRG are integrated into one integrated PIT model by adopting the two medium variables based on their bidirection feedback regulation.

For the corresponding solving algorithm, the proposed PIT model is a multi-objective mixed integer programming that calculate the coordinated optimal solutions for two interacting decision variables (traffic signal scheme and vehicle route plan). Traditional intelligence algorithms (such as heuristics) are low-efficient for solving it due to the dynamic nonlinear relationship between PTSC and PVRG. Inspired by the idea of the game theory [40], a special updating distributed algorithm is designed for solving the PIT model under an asymmetric information-based coordinated framework.

Five typical experimental methods are designed to test the validity and robustness of our contributions by changing certain variables. The results show that: (1) By taking into account the future traffic changes due to other dimensional reconfigurations, all the experiments adopting the isolated improvement in PTSC or PVRG perform better than the existing non-predictive strategies since the future traffic conditions influenced by other traffic dimensions are considered. (2) More than 73.33% of the considered cases adopting the PIT achieve Pareto optimality compared with the isolated improvements in PTSC and PVRG due to the feedback regulation-based iterative optimization. To alleviate traffic congestion, the main contributions of this paper are summarized as follows:

- Compared with the isolated-dimensional TSC strategy, we formulate a PTSC model based on the feedback regulation from VRG whose decisions are considered by introducing a medium variable—predictive traffic flow (determined by the changes in vehicular path plans);
- Compared with the isolated-dimensional VRG strategy, we formulate a PVRG model based on the feedback regulation from TSC whose schemes are considered by introducing a medium variable—predictive vehicular waiting time (determined by different traffic signal schemes);
- We propose a novel “coordinated control” model—PIT for a whole traffic management system based on the two-way feedback regulation between PTSC and PVRG to acquire the coordinated schemes, and design an asymmetric information-based updating distributed algorithm to solve it.

The rest of this paper is organized as follows. Section 2 illustrates the assumptions and formalizations of the art. Section 3 describes the models for alleviating congestion. Section 4 presents the running experiment and comparative results. Conclusions and potential future works are given in Section 5.

## 2. Formalization for This Art

In this section, some preliminary notions are formalized based on general assumptions. The main notations used in this paper are shown in Abbreviations.

### 2.1. Formalization of Traffic Network

**Figure 1:**
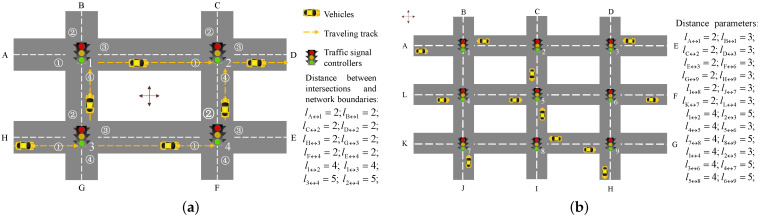
Road networks in a smart city. (**a**) Scenario 1: simple traffic unit, (**b**) Scenario 2: complex traffic network.

For the formalization of the traffic network, this paper assumes typical 4-leg for each intersection and 1-lane (two-way) for each road. Let *j* be an intersection (j∈J) and *i* be a vehicle (i∈I). Let $h_{j,m}^{i}$ denote an optional route node of vehicle *i*, which is the $m_{th}$ entrance of intersection *j* (m∈{1,2,3,4} in 4-leg intersections), $H_{i}=\left\{h_{j,m}^{i}\left|m\in \{1,2,3,4\},\right.j\in J\right\}$ denote the set of optional route nodes of vehicle *i*, and $H=\underset{i\in I}{⋃}H_{i}$ denote the set of optional route nodes of all vehicles. Let $l_{j↔j^{\prime }}$ be the distance between two intersections *j* and $j^{\prime }$. The widths of intersections are ignored in this paper. The classic traffic networks and corresponding distance parameters used in simulation experiments are shown in Figure 1.

In Figure 1, all adjacent intersections are connected by one two-way road. Scenario 1 shows a basic traffic network unit for alternative paths in Figure 1a. Assume all vehicles leave the start point H to destination D. In Figure 1b, it is assumed that each intersection have an original traffic flow towards its farthest diagonal vertex. Scenario 2 shows a complex traffic network of various path combinations (note: the parameters of two scenarios is independent). To study the operating mechanism of our contributions at the micro-level, we consider the two classic traffic scenario to validate in Section 4.

**Figure 2:**
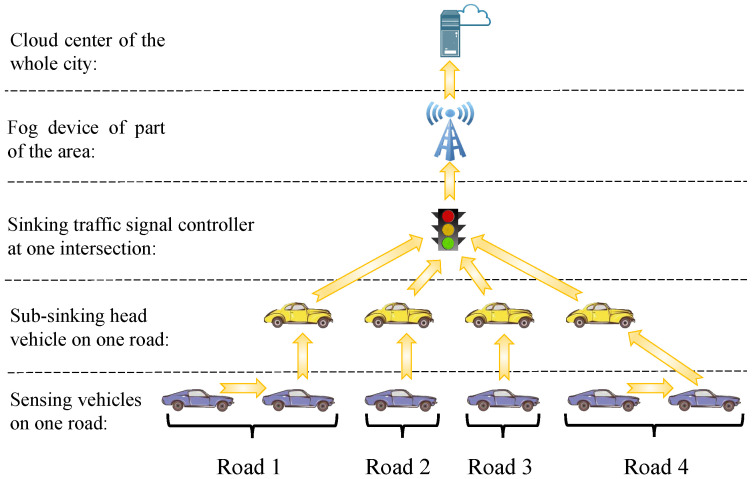
IoVs framework at each intersection *j*.

In this papers’ assumption of intelligent transportation, vehicular route plans and traffic signal schemes can be collected and exchanged by the IoVs [35] and the penetration of vehicles equipped with IoVs devices is 100% [11]. The collecting at each isolated intersection can be assumed in Figure 2, where the information collection protocol is executed periodically as follows:

- Step 1. Based on the V2V technique, all non-head vehicles as sensor nodes send their route plans to the vehicles in front of them until all route information is gathered into the head vehicles (sub-sink nodes) on each road;
- Step 2. Based on the V2I technique, All head vehicles transmit these gathered route plans to the signal controller (sink devices) at intersection;
- Step 3. The sets of route plans and signal schemes are delivered to the cloud center from all signal controllers via the fog devices.

Similarly, the collected path information and signal schemes are delivered from cloud center to edge devices (vehicles and signal controllers) by reverse transmission routing. We assume that there is no case of information deadlock due to excessive distance between mobile nodes (vehicles). Using the assumed IoVs framework, vehicles can receive the signal control schemes at their routes, and signal controllers can collect the vehicular path plans in their control areas.

### 2.2. Formalization of Traffic Signal at Intersections

When a large number of vehicles converge at the same intersections, traffic congestion will occur due to limited traffic capacity. Adjusting the timing or direction of traffic flow elements can avoid vehicular excessive concentration. The existing studies usually adopt TSC or VRG to manage the distribution of traffic flow for reducing traffic congestion.

Traffic signal controllers assign different way leave to entrances of intersection by red and green signals. Let $p_{j,n}$ denote the $n_{th}$ phase of intersection *j*, which is a traffic signal combination of entrances, and *P* be the set of phases of all intersections.

**Figure 3:**
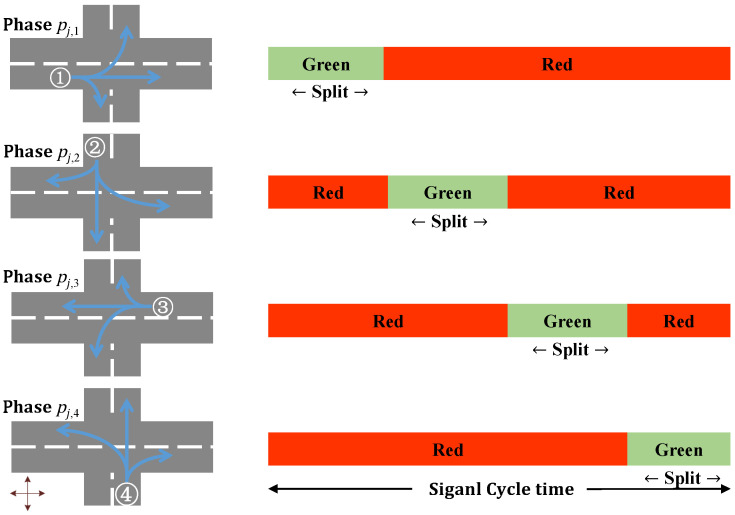
Phases of intersection *j*.

This paper sets four phases (n∈{1,2,3,4}) for signal controllers. Each phase has both green and red signals alternating to regulate traffic flow at intersections (assume that there are no yellow and all-red signals). The vehicles at the red signal entrances have to wait until the green signal, which results in delay time for vehicles. When traffic signals change to green, vehicles need lost time τ (assuming τ=0 in this paper) to restart and speed up. Different signal combinations of entrances at intersection *j* are shown in Figure 3.

In Figure 3, the west flow entrance (m=1) is assigned a green signal and other three entrances are assigned a red signal in $p_{j,1}$ (n=1). In phase $p_{j,2}$ (n=2), the north flow entrance (m=2) is assigned a green signal and other three entrances are assigned a red signal. In phase $p_{j,3}$ (n=3), the east flow entrance (m=3) is assigned a green signal and other three entrances are assigned a red signal. In phase $p_{j,4}$ (n=4), the south flow entrance (m=4) is assigned a green signal and other three entrances are assigned a red signal.

Let $T_{p_{j,n}}^{G}$ denote the green time of phase $p_{j,n}$. The combination of the green time of 4-phases forms a whole signal cycle $T_{j}$ for intersection *j*, i.e.,

$$T_{j}=\sum_{n=1}^{4}T_{p_{j,n}}^{G},\;\forall j\in J.$$

Let $T_{p_{j,n}}^{R}$ denote the red time of phase $p_{j,n}$. We have

$$T_{p_{j,n}}^{R}=T_{j}-T_{p_{j,n}}^{G}.$$

Let $Z_{j}$ be the signal schemes for intersection *j*. Then

$$Z_{j}=\left\{T_{p_{j,n}}^{G}\left|n\in \{1,2,3,4\}\right.\right\},\forall j\in J$$

and $Z=\{Z_{j}\left|j\in J\right.\}$ be the set of signal schemes of all intersections.

When the current signal scheme $Z_{j}\left(t\right)$ is about to end at time *t*, a next signal control scheme $Z_{j}\left(t^{\prime }\right)$ needs to be recalculated and reconfigured at intersection *j*. To avoid the head-vehicles waiting too long and give them enough time to pass intersections, we set the minimum green time $T_{\min}^{G}$ and maximum green time $T_{\max}^{G}$ as done in [16]:

(1) $$T_{\min}^{G}\leq T_{p_{j,n}}^{G}\leq T_{\max}^{G},\;\forall p_{j,n}\in P.$$

TSC releases or blocks traffic flow at intersections by assigning different signals to each phase. For calculating the control schemes $Z_{j}$, vehicular delay time is usually adopted as the evaluation indicator in TSC [8]. Let $\phi_{p_{j,n}}^{i}$ be the delay time of vehicle *i* at the green signal entrances of phase $p_{j,n}$. Take minimum total delay time $\Phi_{j}$

(2) $$\mathrm{Min}\;\Phi_{j}=\sum_{n=1}^{4}\sum_{i\in I}^{}\phi_{p_{j,n}}^{i},\;\forall j\in J$$

as the objective function to generate signal scheme $Z_{j}$ for the isolated intersection *j*. Different traffic signal schemes result in a unique delay time $\phi_{p_{j,n}}^{i}$. For the mapping from $Z_{j}$ to $\phi_{p_{j,n}}^{i}$, based on the assumption that traffic flow is constant, many researchers propose various delay models by theoretical derivation and computer simulation. For example, Webster’s delay model can be simplified as [15]:

(3) $$\phi_{{{}_{p}}_{{}_{j,n}}}^{i}=f\left(Z_{j}\right)$$

where *f* denotes the mapping function from $Z_{j}$ to $\phi_{p_{j,n}}^{i}$. Let $q_{p_{j,n}}$ denote the traffic flow at entrances of phase $p_{j,n}$, $q_{p_{j,n}}^{\max}$ denote the maximum traffic flow at entrances of phase $p_{j,n}$, and AR be the all-red signal time of controller. Webster uses equivalent substitutions and approximate calculations to solve their TSC model and proposes the well-know *Webster Equation*:

(4) $$T_{p_{j,n}}^{G}=\frac{1.5(4\cdot \tau +AR)+5}{1-\sum_{n=1}^{4}\frac{q_{p_{j,n}}}{q_{p_{j,n}}^{\max}}}\cdot \frac{q_{p_{j,n}}}{\sum_{n=1}^{4}q_{p_{j,n}}}$$

that can generate the fixed signal scheme $Z_{j}$ for isolated intersection *j* statically. Due to the limited space, the details of the mathematical derivation are not described in this section.

### 2.3. Formalization of Vehicle Driving

For the formalization of vehicle driving, this paper assumes that vehicles can turn in three directions (going straight, left-turning, and right-turning) at intersections and 100% penetration of on-board computing systems [11]. Let $h_{j,m}^{i}\left(k\right)$ denote the *k*th route node through which vehicle *i* plans to pass. Each vehicle *i* chooses a path direction vector $S_{i}$:

$$S_{i}=\left(h_{j,m}^{i}\left(1\right),h_{j,m}^{i}\left(2\right),\cdots ,h_{j,m}^{i}\left(k\right),\cdots \right),\;\forall i\in I$$

from its current location to destination in the traffic networks of Figure 1. All drivers will follow the suggested routes [41].

Let $\beta_{\mu ,\mu^{\prime }}$ be the path weight from route node μ to another node $\mu^{\prime }$ ($\mu ,\mu^{\prime }\in H$), $A_{i}$ be the vector of estimated arrival time of vehicle *i* arriving at corresponding route nodes $h_{j,m}^{i}$ in $S_{i}$, $R_{i}=\left(S_{i},A_{i}\right)$ be the route plan of vehicle *i*, and $R=\{R_{i}\left|i\in I\right.\}$ be the set of route plans of all vehicles. It means that the vehicle route plan includes the information of path direction and arriving time. We have

$$\left\{\begin{array}{c} A_{i}=\left(t_{i}\left(1\right),t_{i}\left(2\right),\cdots ,t_{i}\left(k\right),\cdots \right)\\ t_{i}\left(k\right)=t_{i}\left(k-1\right)+\beta_{h_{j,m}^{i}\left(k-1\right),h_{j,m}^{i}\left(k\right)} \end{array}\right.,\;\forall i\in I$$

where $t_{i}\left(k\right)$ denotes the predictive arrival time when vehicle *i* arrives at its *k*th route node $h_{j,m}^{i}\left(k\right)$. Path weight $\beta_{h_{j,m}^{i}\left(k-1\right),h_{j,m}^{i}\left(k\right)}$, consisting of free-flow driving time and waiting time at intersections, means the estimated driving time from route node $h_{j,m}^{i}\left(k-1\right)$ to $h_{j,m}^{i}\left(k\right)$.

VRG adjusts the directions of traffic flow by providing recommended paths to drivers. For calculating the route plan $R_{I}$, Vehicular driving time is usually adopted as the evaluation indicator in VRG [19]. Let $x_{\mu ,\mu^{\prime }}^{i}$ denote whether the link from route node μ to a different route node $\mu^{\prime }$ belongs to the path of vehicle *i*. If yes, $x_{\mu ,\mu^{\prime }}^{i}=1$, otherwise, $x_{\mu ,\mu^{\prime }}^{i}=0$. Take minimum driving time $C_{i}$

(5) $$\mathrm{Min}\;C_{i}=\sum_{\mu \in H_{i}}\sum_{\mu^{\prime }\in H_{i}}\beta_{\mu ,\mu^{\prime }}\cdot x_{\mu ,\mu^{\prime }}^{i}$$

as the objective function to calculate route plan $R_{i}$ for vehicle *i*. Different route plans result in a unique set of $x_{\mu ,\mu^{\prime }}^{i}$. For the mapping function *g* from $R_{i}$ to $x_{\mu ,\mu^{\prime }}^{i}$, based on the assumption that path weight is constant, Dijkstra simplifies their nonlinear relation as [20]:

(6) $$x_{\mu ,\mu^{\prime }}^{i}=g\left(R_{i}\right).$$

Take $x_{\mu ,\mu^{\prime }}^{i}$ as decision variable to propose the well-know *Dijkstra Algorithm*:

(7) $$\begin{array}{c} \min\;t_{i}\left(k\right)=\min\{t_{i}\left(k-1\right)+\beta_{\mu ,\mu^{\prime }}\} \end{array}$$

that can select an optimal route direction vector $S_{i}$ for vehicle *i* statically. Due to the limited space and the classic of *Dijkstra Algorithm*, the details of the mathematical derivation are not described in this section.

To improve the management efficiency in the whole intelligent transportation system, the challenges of the integrated model are: (1) the formulation of the nonlinear relationship between TSC and VRG is not easy and (2) designing an effective algorithm to solve the model is not trivial.

## 3. Contributions for Alleviating Traffic Congestion

### 3.1. Motivation

**Figure 4:**
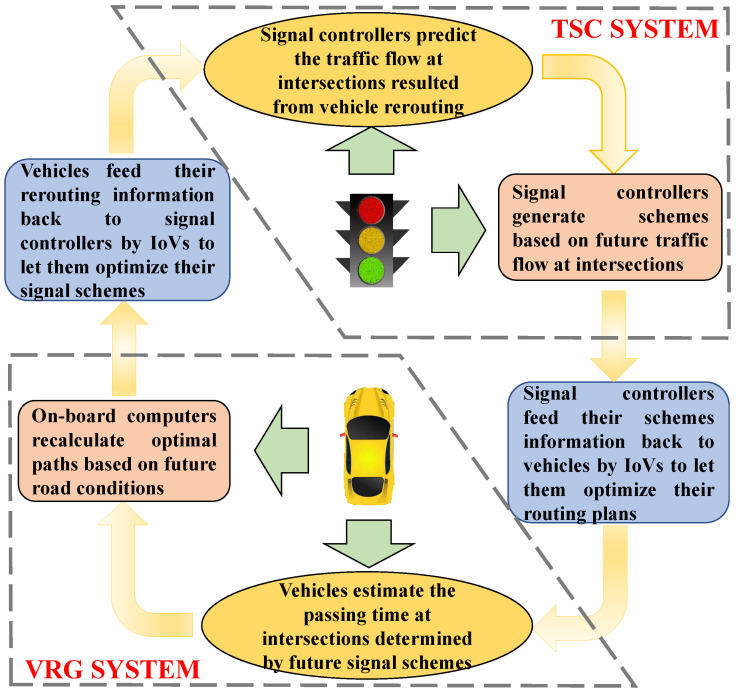
Feedback regulation between TSC and VRG.

Traffic congestion has caused huge unnecessary consumption of urban resources. In real traffic, there exists a two-way feedback regulation between TSC and VRG. As shown in Figure 4, the reconfigurations of vehicle paths could change future distribution of traffic flow. Reversely, different signal control schemes can also determine how long it takes the vehicles to pass through intersections.

In the past, the studies for reducing traffic congestion work solely for TSC or VRG due to the limitation of the communication technologies. The future traffic changes caused by other dimensions’ decisions are largely ignored. In recent years, the emergence of IoVs enables real-time traffic data exchange between different dimensions. Although the works in [38,39] attempt to combine the two dimensions using IoVs technique to alleviate traffic congestion together, their coordinated mechanism ignores the two-way feedback regulation between TSC and VRG. The detailed comparison of related works is shown in Introduction section.

Based on their feedback regulation, we propose the PIT model that is an integration of the PTSC and PVRG to address their respective limitations in this section. To improve this paper’s contributions with the simplest computations, we adopt the most widely known algorithms—*Webster Equation* and *Dijkstra Algorithm* as the benchmark models for PTSC and PVRG to describe our improvements. PTSC and PVRG are the subsystems for PIT. Three contributions are explained in the sequel.

### 3.2. Contribution of PTSC and PVRG

#### 3.2.1. Contribution of PTSC

In TSC, signal controllers assign different signal combinations to each phase to regulate traffic flow. The existing works mainly use advanced IoVs technologies to collect current traffic information to produce signal schemes. In fact, the reconfigurations of vehicle route could change the future distribution of traffic flow that is the supporting data for TSC.

In this section, based on the assumption of 100% penetration of IoVs in Figure 2, we introduce the predictive traffic flow as a medium variable to formulate the link from the varying route plans to the PTSC model. A non-complex future traffic flow prediction algorithm is presented in Algorithm 1 based on dynamic route plans.
**Algorithm 1** Short-term flow prediction for intersections.**Input:** Set of current route directions $S\left(t\right)=\{S_{i}\left(t\right)\left|S_{i}\left(t\right)\right.=(h_{j,m}^{i}\left(1\right),h_{j,m}^{i}\left(2\right),\cdots ,h_{j,m}^{i}\left(k\right),$⋯),i∈I} and set of vehicular estimated arriving time $A\left(t\right)=\{A_{i}\left(t\right)\left|A_{i}\left(t\right)\right.=\left(t_{i}\left(1\right),t_{i}\left(2\right),\cdots ,t_{i}\left(k\right),\cdots \right),i\in I\}$ **Output:** Set of predictive short-term flow $Q\left(t+\Delta t\right)=\{q_{p_{j,n}}\left(t+\Delta t\right)\left|p_{j,n}\right.\in P\}$
  1:**for** $j=1,2,3,...,\left|J\right|$**do**  2:   **for** n=1:4 **do**  3:     **for** $i=1,2,3,...,\left|I\right|$ **do**  4:        **if** the route direction vector $S_{i}\left(t\right)$ of vehicle *i* contains the entrances of phase $p_{j,n}$ **then**  5:          Find the corresponding arrival time $t_{i}\left(k\right)$ of vehicle *i* at this entrance (optional route node)  6:          **if** $t_{i}\left(k\right)\in \left[t,t+\Delta t\right]$ **then**  7:             $q_{p_{j,n}}\left(t+\Delta t\right)=q_{p_{j,n}}\left(t+\Delta t\right)+1$  8:          **end if**  9:        **end if**10:     **end for**11:     Return $q_{p_{j,n}}\left(t+\Delta t\right)$12:   **end for**13:**end for**14:Return Q(t+Δt)


In Algorithm 1, let $q_{p_{j,n}}\left(t+\Delta t\right)$ be the predictive traffic flow at the entrance of phase $p_{j,n}$ during future time Δt. The time complexity of Algorithm 1 is $O(n^{2})$ (note: *n* in time complexity is not the notation of phases). We set $\Delta t=4T_{\max}^{G}$ in this paper since the vehicles arriving at the intersection beyond time $4T_{\max}^{G}$ must not belong to the current signal control cycle. The idea of the flow predictive algorithm is to analyse the arrival time and the corresponding route nodes in route plans. By inputting the set of vehicle route plans R(t), the set of predictive traffic flow Q(t+Δt) is calculated and output by Algorithm 1.

The *Webster Equation* is a classical static signal timing method that is still adopted by the British government to manage traffic flow [8]. In this section, we add the updating predictive traffic flow determined by vehicle rerouting into the traditional *Webster Equation* to formulate the dynamic PTSC model. Let $f_{1}$ be the mapping function from the set of route plans R(t) to the predictive traffic flow Q(t+Δt) in Algorithm 1. The improved constraint conditions of PTSC are as follows:

(8) $$\left\{\begin{array}{c} \phi_{p_{j,n}}^{i}\left(t\right)=f\left(Z_{j}\left(t\right),q_{p_{j,n}}\left(t+\Delta t\right)\right)\\ Q\left(t+\Delta t\right)=f_{1}\left(R\left(t\right)\right)\\ T_{\min}^{G}\leq T_{p_{j,n}}^{G}\left(t\right)\leq T_{\max}^{G}\\ q_{p_{j,n}}\left(t+\Delta t\right)\in Q\left(t+\Delta t\right)\\ T_{p_{j,n}}^{G}\left(t\right)\in Z_{j}\left(t\right) \end{array}\right.$$

Compared with the traditional static constraint conditions of Equation (3) in isolated TSC, Equation (8) implies that the traffic flow is dynamic and the vehicular delay time $\phi_{p_{j,n}}^{i}\left(t\right)$ is caused by the varying route plans R(t) which determine the future traffic flow Q(t+Δt).

Imitating the derivations of the traditional *Webster Equation* (Equation (4)), the improved PTSC model is as follows:

(9) $$\begin{array}{c} T_{p_{j,n}}^{G}\left(t\right)=\left\{\begin{array}{c} T_{\min}^{G}\\ \frac{1.5(4\cdot \tau +AR)+5}{1-\sum_{n=1}^{4}\frac{q_{p_{j,n}}\left(R\left(t\right)\right)}{q_{{}_{p_{j,n}}}^{\max}}}\cdot \frac{q_{p_{j,n}}\left(R\left(t\right)\right)}{\sum_{n=1}^{4}q_{p_{j,n}}\left(R\left(t\right)\right)}\\ T_{\max}^{G}\;\;\;\;\;\;\;\;\;\;\; \end{array}\right. \end{array}$$

In Equation (9), the dynamic signal scheme $Z_{j}\left(t\right)$ is calculated, implying that the route plans determine the future flow ratio between different phases to influence signal schemes. The formulated PTSC model considers the decisions of VRG by introducing a medium variable—predictive traffic flow Q(t+Δt).

#### 3.2.2. Contribution of PVRG

In VRG, a vehicle route guidance system provides recommended paths to drivers to adjust traffic flow trajectories in the space dimension. As the existing works mainly use monitoring devices to detect current traffic conditions to calculate route plans, the future intersection conditions resulting from other dimensions’ decisions, such as TSC, are not considered.

In this section, based on the assumption that all vehicles are equipped with the IoVs in Figure 2, we introduce the predictive vehicular waiting time as a medium variable to formulate the link from different signal schemes to the PVRG model. Let $w_{\mu }$ be the estimated waiting time for vehicle *i* at optional route node μ, and ${\bar{\phi }}_{\mu }$ denote the last delay time of vehicle that has just passed optional route node μ. Based on dynamic signal schemes, a non-complex future waiting time prediction method is formulated as:

(10) $$w_{\mu }\left(t\right)=\max\left\{{\bar{\phi }}_{\mu }\left(t\right),T_{\mu }^{R}\left(Z\left(t\right)\right)\right\},\;\forall \mu \in H$$

where $T_{\mu }^{R}$ denotes the red signal timing of the phase to which the optional route node (entrance) μ belongs. Equation (10) means that the maximum value between ${\bar{\phi }}_{\mu }$ and $T_{\mu }^{R}$ is adopted as the estimated future waiting time of vehicles at node μ. It can avoid the error caused by the waiting for multiple signal cycles. By inputting the set of traffic signal schemes Z(t), the set of estimated waiting time $W\left(t\right)=\left\{w_{\mu }\left(t\right)\left|\mu \right.\in H\right\}$ is generated by Equation (10).

The *Dijkstra Algorithm* is an exact algorithm for static optimal path problems, suitable for the low-dimensional traffic network such as the simulation scenarios in this paper. In this section, we add the updating predictive waiting time determined by the signal controllers’ decisions into the traditional *Dijkstra Algorithm* to formulate the dynamic PVRG model. The improved constraint conditions of PVRG are as follows:

(11) $$\left\{\begin{array}{c} x_{\mu ,\mu^{\prime }}^{i}\left(t\right)=g\left(R_{i}\left(t\right)\right)\\ \beta_{\mu ,\mu^{\prime }}\left(t\right)=l_{j↔j^{\prime }}/v+w_{\mu^{\prime }}\left(t\right)\\ w_{\mu^{\prime }}\left(t\right)=\max\left\{{\bar{\phi }}_{\mu^{\prime }}\left(t\right),T_{\mu^{\prime }}^{R}\left(Z\left(t\right)\right)\right\}\\ x_{\mu ,\mu^{\prime }}^{i}\left(t\right)=0\;\mathrm{or}\;1 \end{array}\right.$$

where *v* denotes the free-flow speed of vehicles, and $l_{j↔j^{\prime }}$ denotes the distance between the corresponding intersections of nodes μ and $\mu^{\prime }$. Compared with the traditional constraint conditions of Equation (6) in isolated VRG, Equation (11) indicates that the vehicle route plan $R_{i}\left(t\right)$ minimizes the vehicular driving time $C_{i}$ in Equation (5) with the red signal timing $T_{\mu^{\prime }}^{R}\left(Z\left(t\right)\right)$ together. Imitating the derivations of Dijkstra [20], we formulate the improved PVRG for vehicle *i* as:

(12) $$\begin{array}{c} \min\;t_{i}\left(k,t\right)=\min\{t_{i}\left(k-1,t\right)+\beta_{\mu ,\mu^{\prime }}\left(Z\left(t\right)\right)\}. \end{array}$$

Equation (12) determines the next optimal route node $\mu^{\prime }$ in vehicle route $S_{i}$ based on minimum arriving time $t_{i}\left(k,t\right)$. By iterating from the start point to destination, the route direction vector $S_{i}\left(t\right)$ for vehicle *i* is generated. The formulated PVRG model considers the decisions Z(t) of TSC by introducing a medium variable—predictive vehicular waiting time W(t).

### 3.3. Contribution of PIT

In a whole intelligent transport system, the distribution of traffic flow is the common results of multi-dimension working together. In this section, we integrate the proposed PTSC and PVRG to formulate a global optimal model—PIT by introducing two medium variables W(t) and Q(t+Δt). Inspired by game theory, we design an asymmetric information exchange framework-based updating distributed algorithm to solve the PIT model.

#### 3.3.1. Model of PIT

Let α be the weight of a single-objective. Based on the feedback regulation between PTSC and PVRG in Figure 4, the proposed PIT model integrated by PTSC and PVRG is:

(13) $$\begin{array}{c} \mathrm{Min}\;(\alpha_{1}\sum_{j\in J}\Phi_{j}\left(t\right)+\alpha_{2}\sum_{i\in I}C_{i}\left(t\right))\\ =\mathrm{Min}\;(\alpha_{1}\sum_{j\in J}^{}\sum_{n=1}^{4}\sum_{i\in I}^{}\phi_{p_{j,n}}^{i}\left(t\right)+\alpha_{2}\sum_{i\in I}^{}\sum_{\mu \in H_{i}}^{}\sum_{\mu^{\prime }\in H_{i}}^{}\beta_{\mu ,\mu^{\prime }}\left(t\right)\cdot x_{\mu ,\mu^{\prime }}^{i}\left(t\right)) \end{array}$$

subject to:

(14) $$\left\{\begin{array}{c} \phi_{p_{j,n}}^{i}\left(t\right)=f\left(Z_{j}\left(t\right),q_{p_{j,n}}\left(t+\Delta t\right)\right)\\ Q\left(t+\Delta t\right)=f_{1}\left(R\left(t\right)\right)\\ T_{\min}^{G}\leq T_{p_{j,n}}^{G}\left(t\right)\leq T_{\max}^{G}\\ q_{p_{j,n}}\left(t+\Delta t\right)\in Q\left(t+\Delta t\right)\\ T_{p_{j,n}}^{G}\left(t\right)\in Z_{j}\left(t\right)\\ x_{\mu ,\mu^{\prime }}^{i}\left(t\right)=g\left(R_{i}\left(t\right)\right)\\ \beta_{\mu ,\mu^{\prime }}\left(t\right)=l_{j↔j^{\prime }}/v+w_{\mu^{\prime }}\left(t\right)\\ w_{\mu^{\prime }}\left(t\right)=\max\left\{{\bar{\phi }}_{\mu^{\prime }}\left(t\right),T_{\mu^{\prime }}^{R}\left(Z\left(t\right)\right)\right\}\\ x_{\mu ,\mu^{\prime }}^{i}\left(t\right)=0\;\mathrm{or}\;1\\ Z_{j}\left(t\right)\in Z\left(t\right)\\ Z_{j}\left(t\right)\in Z\left(t\right) \end{array}\right.$$

where the sets of signal control schemes Z(t) and vehicle route plans R(t) are the decision variables for PIT. The constraint conditions in Equation (14) show that both decision variables have dynamic nonlinear relationship through two medium variables W(t) and Q(t+Δt). The solutions of PIT have the global optimality for a whole traffic management system.

#### 3.3.2. Solving Algorithm for PIT

The proposed PIT model is a multi-objective mixed integer programming that has two interacting decision variables—Z(t) and R(t). Traditional precise algorithms (such as heuristic algorithms) are low-efficient for solving it due to the dynamic nonlinear relationship between decision variables. Inspired by game theory, this section designs an asymmetric information exchange framework-based updating distributed algorithm to approximate the optimal solutions of PIT.

**Figure 5:**
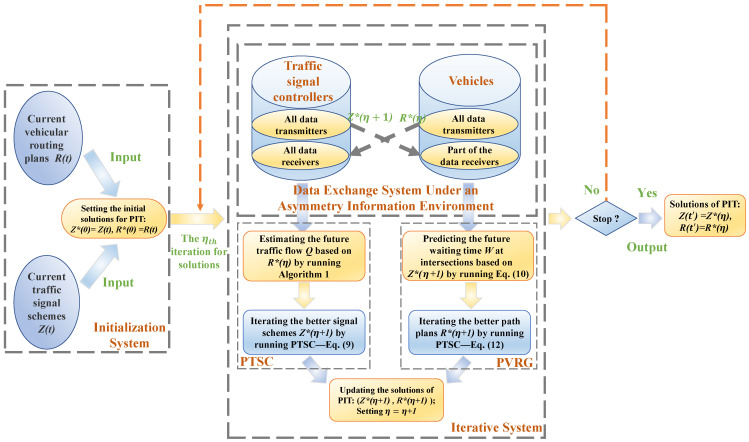
Dynamic game processes and rules in the updating distributed algorithm.

A complete game model includes four basic elements—players, strategies, payoff functions, and game rules. Each player tries to improve its payoff by determining an optimal strategy based on the game rules. In our updating distributed algorithm, the set of vehicles *I* and set of intersections *J* are two players. The selectable paths and signal control schemes are strategy sets for vehicles and intersections, respectively. The payoff functions of players *I* and *J* are the total vehicular driving time *C* and delay time Φ, respectively. It should be noted that a symmetric information exchange framework could result to the “route flapping” phenomenon where a congestion switches from one road to an alternative road when numerous vehicles obey a same guidance [38]. The asymmetric information exchange framework-based dynamic game processes and rules are shown in Figure 5.

In the designed dynamic game processes of Figure 5, in the fact, we decompose the interacting multi-objectives function Equation (13) to:

(15) $$\begin{array}{c} \alpha_{1}\sum_{j\in J}\mathrm{Min}\;\sum_{n=1}^{4}\sum_{i\in I}^{}\phi_{p_{j,n}}^{i}\left(t\right)+\alpha_{2}\sum_{i\in I}\mathrm{Min}\sum_{\mu \in H_{i}}\sum_{\mu^{\prime }\in H_{i}}\beta_{\mu ,\mu^{\prime }}\left(t\right)\cdot x_{\mu ,\mu^{\prime }}^{i}\left(t\right) \end{array}$$

where only the single-objective $Z_{j}\left(t\right)$ of PTSC and $R_{i}\left(t\right)$ of PVRG need to be calculated and pursued by players. We set intersections as the dominant players in the game. The updating sequence of the distributed agents’ schemes is like a difference equation:

$$R^{*}\left(\eta \right)\to Z^{*}\left(\eta +1\right)\to R^{*}\left(\eta +1\right)\to Z^{*}\left(\eta +2\right)\to \cdots$$

where signal controllers receive all vehicles’ virtual plans but only transmit their attempts to part of the vehicles. By receiving each other’s virtual decisions constantly, the signal controllers and vehicles iterate their better virtual plans. They constantly exchange their last virtual schemes in an asymmetry information environment, which can reduce the “route flapping” phenomenon by preventing vehicles to divert themselves to the same alternative roads simultaneously. The pseudo-code of our updating distributed algorithm compiled from Figure 5 is shown in Algorithm 2.
**Algorithm 2** Pseudo-code of our updating distributed algorithm for solving proposed PIT model.**Input:** Sets of current signal control schemes Z(t) and current vehicular route plans R(t) **Output:** Sets of next signal control schemes $Z(t^{\prime })$ of PTSC and next vehicle route plans $R(t^{\prime })$ of PVRG  1:Set the current schemes as the initial value: $Z^{*}\left(0\right)=Z\left(t\right);\;R^{*}\left(0\right)=R\left(t\right)$  2:**for** $\eta =0,1,2,\cdots ,\eta_{0}$ **do**  3:   Vehicles transmit their last virtual route plans $R^{*}\left(\eta \right)$ to all signal controllers based on the IoVs framework in Figure 2  4:   Execute Algorithm 1 to predict traffic flow $Q(t+4T_{\max}^{G})$ at intersections  5:   Execute PTSC model (Equation (9)) to update the virtual signal control schemes $Z^{*}\left(\eta +1\right)$  6:   Signal controllers transmit their last virtual schemes $Z^{*}\left(\eta +1\right)$ to part of the vehicles based on the IoVs framework in Figure 2  7:   Execute Equation (10) to estimate the vehicular waiting time W(t) at intersections  8:   Execute PVRG model (Equation (12)) to update the virtual route plans $R^{*}\left(\eta +1\right)$  9:   Let η=η+1 and decide whether to stop iteration.10:**end for**11:Return the set of next schemes of PIT: $Z\left(t^{\prime }\right)=Z^{*}\left(\eta \right);\;R\left(t^{\prime }\right)=R^{*}\left(\eta \right)$


In Algorithm 2, since there is not a proof to guarantee the convergence of the updating distributed algorithm, the experiments choose other stop criteria such as the maximum iteration times $\eta_{0}$ in each cycle. The output of the designed updating distributed algorithm is an approximate solutions for PIT compared with the precise global optimality. The time complexity of Algorithm 2 is $O(n^{3})$.

### 3.4. Discussion

The existing IoVs-based methods for alleviating traffic congestion only use real-time traffic data to produce decisions in isolated dimension control of TSC or VRG, which results in the waste of city resources due to the ignorance of influence from other traffic dimensions. In this section, by introducing two medium variables—predictive traffic flow and predictive vehicular waiting time, we formulate a PIT model based on the two-way feedback regulation between TSC and VRG to alleviate traffic congestion by generating the coordinated reconfiguration schemes for multi-dimension together. Moreover, an asymmetric information exchange framework-based updating distributed algorithm is designed to solve the PIT based on the idea of the game theory, which can reduce the “route flapping” phenomenon by preventing numerous vehicles to divert themselves to the same alternative roads simultaneously.

## 4. Simulation Experiments

The performance of the PIT and the corresponding updating distributed algorithm is hard to be studied by rigorous mathematical discussion. In this section, simulation experimental methods (shown in Table 1) are designed based on the single-variable control (each experimental condition varies in turn) to verify the validity of our contributions. They are run on the typical scenarios in Figure 1 to test the validity and robustness of the experimental variables—PTSC, PVRG, and PIT. The MATLAB R2016a tool is used to compile one vehicular driving environment in which information between PTSC and PVRG is constantly exchanged. The pseudo-code of the experiments is shown in Algorithm 3 (the time complexity of Algorithm 3 is $O(n^{4})$).
**Algorithm 3** Pseudo-code for vehicles driving in the experiments.**Input:** Set of initial configuration schemes of signal control Z(0) and the set of initial configuration plans of vehicle route R(0) **Output:** Set of intersections’ delay time $\Phi =\{\Phi_{j}\left|j\right.\in J\}$ and the set of vehicular driving time $C=\{C_{i}\left|i\right.\in I\}$  1:**for** t=1,2,3,…, **do**  2:   Vehicles that do not arrive intersections move forward one unit on the traffic grid  3:   Vehicles queuing in front of the green signal move forward one unit on the traffic grid  4:   **if** All experimental vehicles arrive their destinations **then**  5:     BREAK  6:   **end if**  7:   **if** Existing signal controllers are about to end their current schemes **then**  8:     Generating and configuring next schemes (using the PIT model and Algorithm 2) for signal controllers and vehicles  9:   **end if**10:   Setting t=t+111:**end for**12:Return the set of intersections’ delay time Φ and set of vehicular driving time *C*


### 4.1. Preparation

For the common parameters of experiments in Table 1, the $T_{\min}^{G}$ and $T_{\max}^{G}$ are set to 1 and 5 time-units, respectively, except for intersection 3 of Scenario 1. The $T_{\max}^{G}$ of intersection 3 in Scenario 1 is increased to 15 time-units to ensure sufficient flow for downstream intersections due to the unbalanced flow distribution. The maximum traffic flow of phases is $q_{p_{j,n}}^{\max}\;=\;1\;(\mathrm{veh}/\mathrm{time}-\mathrm{unit})$. The all-red signal time AR=0 and the vehicle speed v=1 in this paper [42]. Vehicles change their route plans after the traffic signal has been reconfigured. To eliminate the interference from other irrelevant factors, this paper chooses the most concise comparison experiments (shown in Table 1) to verify the three contributions:

For method 1 in Table 1, the average time $(T_{\min}^{G}+T_{\max}^{G})/2$ is adopted as signal timing in the fixed TSC strategies, where do not exists the feedback regulation to vehicle routing due to the invariable signal schemes. The corresponding VRG system considers only free-flow driving time calculated by constant distance parameters to generate vehicle routes.

For method 2 in Table 1, the dynamic TSC is added into method 1. The current vehicular number on the road is collected and used to generate dynamic signal schemes without considering the future vehicles coming into this road. By compared to the fixed TSC in method 1, the feasibility of the basic dynamic TSC (referenced model for PTSC) can be analyzed.

For method 3 in Table 1, the dynamic function is added into the fixed VRG of method 2 by predicting waiting time at intersections using changing signal schemes. Their detailed calculations are described in Section 3.2.2. By comparing with the non-predictive fixed VRG in method 2, the feasibility of our proposed PVRG which considers the feedback regulation from the dynamic traffic signal schemes can be verified.

For method 4 in Table 1, the predictive function is added into the dynamic TSC of method 3, which means the future traffic flow regulated from VRG is evaluated and used for PTSC. Their detailed calculations are described in Section 3.2.1. Compared with the non-predictive dynamic TSC in method 3, the feasibility of our proposed PTSC which considers the feedback regulation from vehicle rerouting can be demonstrated. It should be noted that although the two dimensions (PVRG and PTSC ) work together in method 4, there is no iterating and updating for the better coordinated schemes based on their feedback regulation relationship.

For method 5, the asymmetric information exchange framework-based coordinated mechanism between PTSC and PVRG in PIT is added into the non-iterative method 4. The detailed calculations are shown in Section 3.3. To simplify calculations, the maximum iteration times $\eta_{0}$ in Algorithm 2 is set to a fixed value 10. The coefficient of asymmetric information is set to a fixed value 0.5, implying that 50% of the vehicles can receive the virtual signal schemes randomly in each iteration. Compared with the non-coordinated dynamic PTSC and PVRG in method 4, the feasibility of our designed PIT can be concluded which uses the two-way feedback regulation to iterate coordinated schemes for PTSC and PVRG.

### 4.2. Validity Test and Sensitivity Analysis

#### 4.2.1. Validity Test

Scenario 1 in Figure 1 is a basic traffic network unit for an alternative path. The feasibility of the contributions at the micro-level can be tested by running the above five methods on Scenario 1. Let 150 vehicles leave the start point H to destination D with 1veh/(unit-time) in Scenario 1. That means only two paths can be selected for each vehicle *i* (Path 1: $H\to h_{3,1}^{i}\to h_{1,4}^{i}\to h_{2,1}^{i}\to D$; Path 2: $H\to h_{3,1}^{i}\to h_{4,1}^{i}\to h_{2,4}^{i}\to D$). The results are shown in Table 2 and Table 3.

As shown in Table 2 and Table 3, we can see that:

(1) The total driving time and delay time of method 2 have been decreased by 51.01% and 69.51%, respectively, compared with method 1 due to the working of dynamic TSC strategy. It proves the effectiveness of the non-predictive dynamic TSC model.

(2) Comparing the results of method 3 with 2, the total driving time and delay time of method 3 have been decreased by 6.35% and 15.19%, respectively. About 56 vehicles divert to path 2 due to the working of predictive rerouting in PVRG which can consider the future waiting time (influenced from the dynamic TSC schemes) at intersections. The potential of the built PVRG have been demonstrated to improve traffic efficiency.

(3) Comparing the results of method 4 with 3, the total driving time and delay time of method 4 have been decreased by 2.25% and 6.64%, respectively, due to the working of the predictive function in PTSC model which. It shows that the function of the built PTSC is effective since that the future traffic flow determined by the dynamic route information is considered.

(4) Compared with the non-coordinated mechanism between PTSC and PVRG (method 4), the solutions of our PIT (method 5) reduce the driving time and delay time by 2.22% and 5.08%, respectively, due to the asymmetric information exchange framework-based coordinated mechanism. The asymmetric information-based coordinated between PTSC and PVRG can reduce the “route flapping” phenomenon by preventing numerous vehicles to divert themselves to the same alternative roads simultaneously. The feasibility of our PIT model is verified.

As the above analysis in simulation experiments, the built PTSC and PVRG introduce medium variables—predictive traffic flow and vehicular waiting time, respectively, to consider each other’s feedback regulation to achieve “Prediction” function in further improving traffic efficiency. Furthermore, compared with the isolated improvement in PTSC and PVRG, the asymmetric information exchange framework-based coordinated mechanism in PIT achieve Pareto optimization for the whole traffic management system due to the reducing of “route flapping” phenomenon by preventing.

#### 4.2.2. Sensitivity Analysis

**Figure 6:**
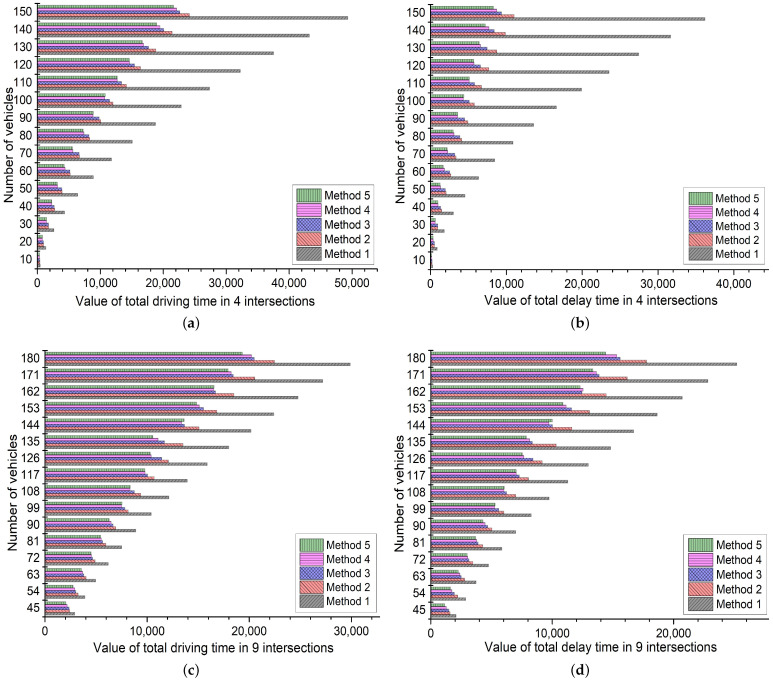
Sensitivity analysis for the results. (**a**) Total driving time of vehicles in 4 intersections, (**b**) Total delay time of signal controllers in 4 intersections, (**c**) Total driving time of vehicles in 9 intersections, (**d**) Total delay time of signal controllers in 9 intersections.

To avoid the contingency of special parameter setting, this paper changes the numbers of vehicles and intersections to test the robustness of the above results. First the vehicle number is changed from 50 to 200 in Scenario 1. Then the vehicle number at each start point is changed from 5 to 20 in Scenario 2 where the number of intersections is increased to 9 compared with Scenario 1. We assume that the starting points and corresponding destinations are as follows: A→G, C→F, D→J, L→I, $h_{5,1}^{i}\to H$, F→C, K→D, I→L, H→B in Figure 1b. The results of sensitivity analysis are shown in Figure 6. For economy of space, the detailed data are not presented in this section. Some remarkable findings are discovered:

(1) In Figure 6a,b, for the robustness of the non-predictive dynamic TSC model, method 2 still performs better than method 1 with the increased vehicle number in Scenario 1. The average improvement efficiency is 47.85% and 65.81% in driving time and delay time, respectively. For the robustness of PVRG, all experiments in method 3 perform better than method 2 due to the working of predictive rerouting. The average improvement efficiency is 4.69% and 11.82% in driving time and delay time, respectively. For the robustness of PTSC, all experiments in method 4 achieve better results than method 3 due to the prediction of future traffic flow in TSC. The average improvement efficiency is 6.63% and 15.21% in driving time and delay time, respectively. For the robustness of PIT, the overall performance of method 5 remains better than the isolated improvements in PTSC and PVRG due to the function of our coordinated mechanism. Compared with method 4, 73.33% of the experiments obtain better results. The average improvement efficiency is 1.18% and 2.70% in driving time and delay time, respectively.
(2) In Figure 6b,c, similarly, the functions of non-predictive dynamic TSC model, PVRG, PTSC and PIT are still conspicuous with the increased number of intersections in Scenario 2. Specifically, the average improvement efficiencies in driving time and delay time are 23.75% and 29.04% (comparing method 2 with 1), 8.00% and 11.97% (comparing method 3 with 2) 2.99% and 3.30% (comparing method 4 with 3), and 1.56% and 2.20% (comparing method 5 with 4), respectively. The efficiency of all methods is consistent with the conclusions in Section 4.2.1.

As for the above analysis, the feasibility of PTSC, PVRG, and PIT still hold by changing the numbers of vehicles and intersections in the sensitivity analysis. Equation (10) leads to the main error in simulations since this formula cannot accurately predict signal color when vehicles arrive at intersections.

### 4.3. Discussion

In this section, by controlling single-variable (PVRG, PTSC, PIT) and comparing them with existing major strategies, the results can be summarized as follows: (1) The improvement in isolated PVRG is consistently better than the non-predictive VRG strategy due to the consideration of future vehicular waiting time at intersections determined by TSC. (2) The improvement in isolated PTSC is consistently better than the non-predictive TSC strategy due to the consideration of future traffic flow caused by VRG. (3) The solutions to PIT can achieve Pareto optimality compared with isolated PTSC and PVRG by constantly exchanging their future virtual schemes based on their two-way feedback regulation. These conclusions still hold by changing the numbers of vehicles and intersections in the sensitivity analysis.

## 5. Conclusions

This paper formulates the PTSC and PVRG models that consider future traffic changes caused by other dimensions’ decisions, and integrates them into an integrated PIT model (whose solutions are coordinated optimal in the whole traffic management system) based on their feedback regulation relationship. Moreover, inspired by game theory, an asymmetric information exchange framework-based updating distributed algorithm is designed to solve the PIT model, which can prevent vehicles diverting to the same alternative roads simultaneously to alleviate the “route flapping” phenomenon in iterative optimization. Through the experiments we show that the formulated PVRG and PTSC consistently perform better than the non-predictive strategies since the future traffic conditions influenced by other traffic dimensions are considered. The solutions of PIT achieve Pareto optimality compared with the isolated improvements in PTSC and PVRG due to the working of their feedback regulation. These conclusions also have good robustness in the sensitivity analysis.

## Figures and Tables

**Table 1:** Methods of simulation experiments.

Methods	Explanation	Purpose
Method 1: Fixed TSC + Fixed VRG	Signal controllers adopt the fixed timing strategies; Vehicles calculate routes based on constant distance parameters	Control group
Adding non-predictive dynamic TSC into Method 1		
Method 2: Dynamic TSC + Fixed VRG	Signal controllers generate dynamic signal schemes based only on current traffic flow on their roads; Vehicles calculate routes based on constant distance parameters	Verifying the feasibility of non-predictive dynamic TSC
Adding the proposed PVRG into Method 2 (for example the work in [43] adopts the predictive vehicle reroute strategy to alleviate traffic congestion)		
Method 3: Dynamic TSC + PVRG	Signal controllers adopt the non-predictive dynamic TSC strategies; Vehicles reroute considering predictive waiting time influenced from dynamic TSC schemes (PVRG in Section 3.2.2)	Verifying the feasibility of the proposed PVRG
Adding the proposed PTSC into Method 3 (for example the work in [6] adopts the predictive signal control strategy to alleviate traffic congestion)		
Method 4: PTSC + PVRG without the coordinated mechanism of PIT	Signal controllers generate the dynamic schemes considering future traffic flow determined by dynamic VRG (PTSC in Section 3.2.1); Vehicles reroute based on the proposed PVRG	Verifying the feasibility of the proposed PTSC
Adding the feedback regulation-based coordinated control framework of the proposed PIT model into Method 4		
Method 5: PIT	PTSC and PVRG iterate together based on an asymmetric information exchange environment in Section 3.3	Verifying the feasibility of this paper’s PIT model and the solving algorithm

**Table 2:** Total driving time of vehicles.

Methods	Path 1	Path 2	Total Driving Time	Efficiency
Method 1	150	0	49,315	0
Method 2	150	0	24,159	51.01%
Method 3	94	56	22,626	54.12%
Method 4	51	99	22,117	55.15%
Method 5	71	79	21,626	56.15%

Note: The efficiency column denotes the percentages of improvement compared to method 1.

**Table 3:** Total delay time of intersections.

Methods	Entrance $h_{1,4}^{i}$	Entrance $h_{2,1}^{i}$	Entrance $h_{2,4}^{i}$	Entrance $h_{3,1}^{i}$	Entrance $h_{4,1}^{i}$	Total	Efficiency
Method 1	2431	441	0	33,318	0	36,190	0
Method 2	4767	1329	0	4938	0	11,034	69.51%
Method 3	1575	1092	856	4938	897	9358	74.14%
Method 4	498	249	1285	4944	1761	8737	75.86%
Method 5	818	336	1028	4944	1167	8293	77.08%

Note: The efficiency column denotes the percentages of improvement compared to method 1.

## Data Availability

The data presented in this study are available on request from the corresponding author. The data are not publicly available due to privacy.

## Abbreviations

PIT	Predictive intelligent transportation
IoVs	Internet of Vehicles
(P)TSC	(Predictive) Traffic signal control
(P)VRG	(Predictive) Vehicle routing guidance
*I*	Set of vehicles
*J*	Set of intersections
$l_{j↔j^{\prime }}$	Distance between two different intersections *j* and $j^{\prime }$
$h_{j,m}^{i}$	Optional route node of vehicle *i* at the mth entrance of intersection *j*
$H_{i}$	Set of optional route nodes of vehicle *i*, $H_{i}=\left\{h_{j,m}^{i}\left|m\in \{1,2,3,4\},\right.j\in J\right\}$
*H*	Set of optional route nodes of all vehicles, $H=\underset{i\in I}{⋃}H_{i}$
$\beta_{\mu ,\mu^{\prime }}$	Path weight from route node μ to a different route node $\mu^{\prime }$, $\mu ,\mu^{\prime }\in H$
$w_{\mu }$	Estimated waiting time of vehicles at route node μ
*W*	Set of estimated waiting time, $W=\{w_{\mu }\left|\mu \right.\in H\}$
$R_{i}$	Route plan of vehicle *i*
*R*	Set of vehicle route plans, $R=\{R_{i}\left|i\in I\right.\}$
$C_{i}$	Driving time of vehicle *i*
$p_{j,n}$	nth phase of intersection *j*, n∈{1,2,3,4} in this paper
*P*	Set of phases, $P=\{p_{j,n}\left|n\in \{1,2,3,4\},\right.j\in J\}$
$q_{p_{j,n}}$	Estimated traffic flow at the entrances of phase $p_{j,n}$
*Q*	Set of estimated traffic flow, $Q=\{q_{p_{j,n}}\left|p_{j,n}\in P\right.\}$
$T_{j}$	Traffic signal cycle of intersection *j*, $T_{j}\in Z^{+}$
$T_{p_{j,n}}^{G}$	Green traffic signal of phase $p_{j,n}$, $T_{p_{j,n}}^{G}\in Z^{+}$
$T_{p_{j,n}}^{R}$	Red traffic signal of phase $p_{j,n}$, $T_{p_{j,n}}^{R}\in Z^{+}$
$Z_{j}$	Traffic signal scheme of intersection *j*
*Z*	Set of traffic signal schemes, $Z=\{Z_{j}\left|j\in J\right.\}$
$\phi_{p_{j,n}}^{i}$	Delay time of vehicle *i* at the green signal entrances of phase $p_{j,n}$
$\Phi_{j}$	Total vehicular delay time of intersection *j*
$Z^{*}$	Set of virtual schemes of traffic signal
$R^{*}$	Set of virtual plans of vehicle route
